# A Novel Approach to Reinstating Tolerance in Experimental Autoimmune Myasthenia Gravis Using a Targeted Fusion Protein, mCTA1–T146

**DOI:** 10.3389/fimmu.2017.01133

**Published:** 2017-09-13

**Authors:** Alessandra Consonni, Sapna Sharma, Karin Schön, Cristina Lebrero-Fernández, Elena Rinaldi, Nils Yngve Lycke, Fulvio Baggi

**Affiliations:** ^1^Neurology IV, Fondazione I.R.C.C.S. Istituto Neurologico Carlo Besta, Milan, Italy; ^2^Department of Microbiology and Immunology, Institute of Biomedicine, University of Gothenburg, Gothenburg, Sweden

**Keywords:** tolerance, adjuvant, vaccination, dendritic cells, myasthenia gravis, autoimmune disease, cholera toxin

## Abstract

Reinstating tissue-specific tolerance has attracted much attention as a means to treat autoimmune diseases. However, despite promising results in rodent models of autoimmune diseases, no established tolerogenic therapy is clinically available yet. In the experimental autoimmune myasthenia gravis (EAMG) model several protocols have been reported that induce tolerance against the prime disease-associated antigen, the acetylcholine receptor (AChR) at the neuromuscular junction. Using the whole AChR, the extracellular part or peptides derived from the receptor, investigators have reported variable success with their treatments, though, usually relatively large amounts of antigen has been required. Hence, there is a need for better formulations and strategies to improve on the efficacy of the tolerance-inducing therapies. Here, we report on a novel targeted fusion protein carrying the immunodominant peptide from AChR, mCTA1–T146, which given intranasally in repeated microgram doses strongly suppressed induction as well as ongoing EAMG disease in mice. The results corroborate our previous findings, using the same fusion protein approach, in the collagen-induced arthritis model showing dramatic suppressive effects on Th1 and Th17 autoaggressive CD4 T cells and upregulated regulatory T cell activities with enhanced IL10 production. A suppressive gene signature with upregulated expression of mRNA for TGFβ, IL10, IL27, and Foxp3 was clearly detectable in lymph node and spleen following intranasal treatment with mCTA1–T146. Amelioration of EAMG disease was accompanied by reduced loss of muscle AChR and lower levels of anti-AChR serum antibodies. We believe this targeted highly effective fusion protein mCTA1–T146 is a promising candidate for clinical evaluation in myasthenia gravis patients.

## Introduction

Myasthenia Gravis (MG) is an autoimmune disease characterized by muscle weakness and fatigability, which, in most cases, is the result of autoantibody production against the acetylcholine receptor (AChR) at the neuromuscular junction (1, 2). Although the disease is strongly associated with autoantibody production, the AChR-specific CD4^+^ T cells have a central regulatory role and, indeed, also patients lacking anti-AChR antibodies host peripheral blood CD4^+^ T cells that react to AChR-peptides (3, 4). Importantly, MG patients have reduced levels of regulatory T cells (Tregs) (5–8) and restoring the levels of Tregs in the experimental autoimmune myasthenia gravis (EAMG) model suppressed disease (9). Hence, reinstating a functional Treg population could be a curative therapeutic intervention in MG (10). Both Th1 and Th17 cells have been implicated in driving the autoimmune attack, but the precise contribution of the respective subset remains unclear (11–13). Evidence in support of an involvement of Th17 cells in the EAMG model has been documented in several recent studies, while earlier studies, for example, in Tbet^−/−^ mice demonstrated reduced susceptibility to EAMG as a consequence of fewer autoreactive Th1 cells (14).

Current treatments for autoimmune diseases are not curative, but rather are directed against the immunopathology and symptoms (15, 16). Also in MG, the first line of treatment is symptomatic, counteracting the muscle fatigability using acetylcholinesterase inhibitors, while some patients need additional medication with steroids or other forms of immunosuppressants, such as azathioprine, mycophenolate, or methotrexate. In the more severe cases, monoclonal antibodies such as anti-CD20 (rituximab) or, for short-term benefit, intravenous immunoglobulin or even plasmapheresis/immunoadsorption are being used (17). The medication has to be taken life-long with an increased risk of acquiring infectious diseases or even cancer, secondarily to severe side effects (15). Therefore, there is growing interest in developing novel therapies able to suppress autoaggressive T cells and reinstate tolerance in the immune system (18).

One such approach is to use adoptive cell transfer of *ex vivo* expanded autologous CD4^+^ Treg cells to inhibit disease development as has been successfully demonstrated in the EAMG model (9). Other investigators have focused on identifying immunodominant epitopes in the AChR to raise Tregs by immunization and in this way protect against disease development (19). Thus, restoring a functional Treg population by immunization with immunodominant epitopes from the AChR appears to be an attractive therapeutic approach for curbing MG disease. A major challenge, though, has been to translate the very promising findings in the rodent EAMG models into effective immunization protocols for treatment of MG patients (20, 21). This could partly be explained by the lack of effective formulations for tolerance-induction in humans as both disease-relevant peptides and proteins have already been identified but clinical testing still awaits to be done (18, 22–25). Hence, we need better ways of formulating our candidate epitopes to secure a strong induction of tolerance also in the clinic.

We have developed CTA1R9K-X-DD, which is a targeted immunomodulating fusion protein that can carry different disease-relevant peptides and which is an effective tolerogenic vector for the suppression of autoaggressive CD4 T cells (26). The fusion protein is an inactivated mutant of the CTA1 subunit of cholera toxin, which in its native form exerts strong ADP-ribosylating effects (26). The DD is a dimer of a fragment of *Staphylococcus aureus* proteinA, which targets classical dendritic cells (27). Using the collagen-induced arthritis (CIA) mouse model, we could demonstrate that a collagen peptide (aa 259–274) inserted into the fusion protein, CTA1R7K-COL_259–274_-DD, and given as an intranasal (i.n.) therapy or orally in the form of edible plants effectively protected against CIA (26, 27). Following treatment with CTA1R7K-COL_259–274_-DD, we found suppression of specific antibody levels in serum, reduced effector Th1 and Th17 CD4 T cell responses to peptide concomitant with an increased production of IL-10, while IL-6 and MMP3 levels were strongly reduced (26, 27). Although we observed increased numbers of circulating Foxp3^+^ Tregs, the increased IL-10 production emanated from regulatory Foxp3^−^ CD4 T cells, i.e., Tr1-like cells (28).

In the present study, we have extended our work to the EAMG model. We hypothesized that the mechanism of action in the EAMG model could be the induction of Tr1 cells and the subsequent reinstatement of tolerance to the AChR. Therefore, a disease-relevant peptide, the 146–162 amino acid peptide from AChR, was expressed as a fusion protein, CTA1R9K-AChR_146–162_-DD (hereinafter referred as mCTA1–T146). This fusion protein, thus, carried a dominant epitope from the AChR for treatment of EAMG in C57Bl/6 mice (29, 30). We report here the successful use of this fusion protein in both acute and chronic stages of EAMG disease. Treated mice developed significantly less symptoms and exhibited less tissue destruction and lower serum anti-AChR antibody titers. Lymph node cells in treated mice demonstrated upregulated gene expression of TGFβ, IL10, IL27, and Foxp3, a suppressive gene signature, concomitant with suppressed Th1 and Th17 CD4^+^ T cell development (31, 32).

## Results

### Intranasal Treatment Suppresses CD4^+^ T Cell Priming in the EAMG Model

**Figure 1:**
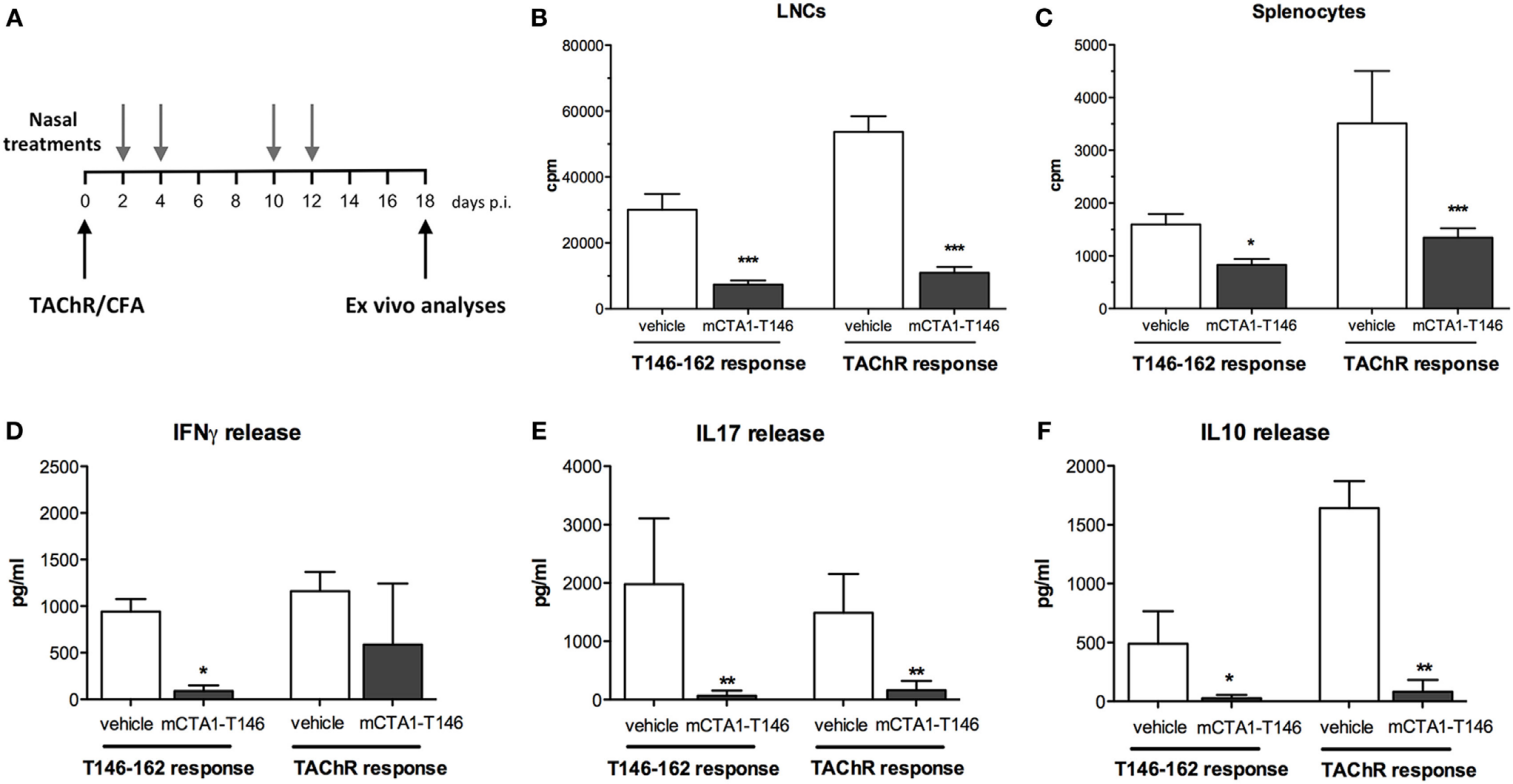
Intranasal mCTA1–T146 treatment strongly suppresses CD4^+^ T cell priming. **(A)** Experimental design: a single s.c. injection of TAChR at the base of the tail followed by four nasal treatments with mutant CTA1R9K-AChR_146_-DD (mCTA1–T146) (5 µg/dose) or PBS (vehicle). On day 18, popliteal and inguinal LNCs **(B)** and splenocytes **(C)** were analyzed for proliferation to recall antigen TAChR protein (0.25 µg/ml) or T146–162 peptide (10 µg/ml). After 72 h, supernatants from cultured LNCs were evaluated for IFNγ **(D)**, IL17 **(E)**, and IL10 **(F)** cytokine release using multiparametric assay (Luminex). Representative experiment of two; *n* = 5 mice per group. Data represent means of mice analyzed individually with isolated cells in triplicate cultures and given as mean cpm ± SEM **(B,C)** or pg/ml ± SD **(D–F)**. Significance was calculated with Mann–Whitney test. *p*-Values are represented by *p < 0.05, **p < 0.01, and ***p < 0.001.

Previous studies in the CIA model suggested that immune tolerance could also be achieved in other models of autoimmune diseases, provided disease-relevant peptides were incorporated in the CTA1R9K-X-DD fusion protein (26). Therefore, we designed, expressed, and purified a fusion protein, mCTA1–T146, which carried a dominant epitope corresponding to the aa 146–162 sequence from the alpha subunit of the torpedo AChR (30) for treatment of disease in the EAMG mouse model (21, 33). In order to study the effect of the fusion protein, TAChR-primed mice were given the fusion protein mCTA1–T146 or PBS i.n. on days 2, 4, 10, and 12 after the s.c. immunization. Mice were sacrificed on day 18 and the immune response to TAChR was evaluated in single cell suspensions from the spleen and draining (inguinal and popliteal) lymph nodes (Figure 1A). We observed a striking suppression of the CD4^+^ T cell recall response to both TAChR and T146–162 peptide in the draining lymph nodes and spleen in mice treated with the mCTA1–T146 fusion protein, while PBS-treated mice showed robust responses (Figures 1B,C). In particular, CD4^+^ T cell proliferation in the draining lymph nodes and spleen was reduced by 70–80% (Figures 1B,C). The lower recall responses were also reflected in poor cytokine production, with dramatically reduced IFNγ, IL17, and IL10 levels in culture supernatants from mCTA1–T146 treated as compared to PBS-treated mice (Figures 1D–F).

### The mCTA1–T146 Fusion Protein Protects against EAMG Disease

**Figure 2:**
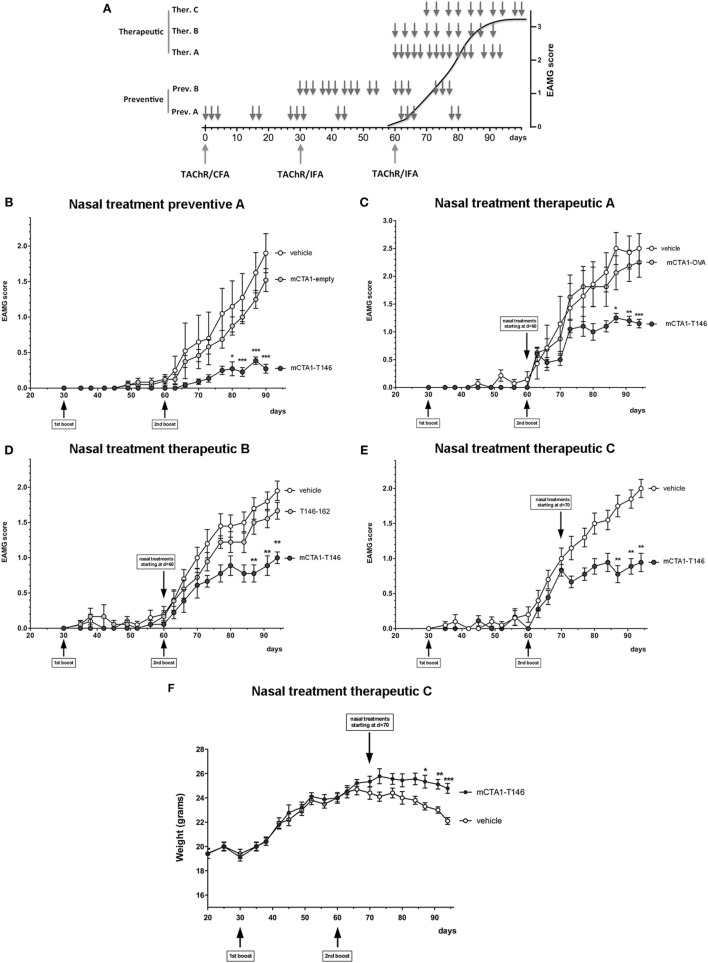
Intranasal treatment with mCTA1–T146 protects against experimental autoimmune myasthenia gravis (EAMG) disease. **(A)** Treatment protocols evaluated in the mouse EAMG model. Animals were i.n. treated with CTA1R9K-AChR_146_-DD (mCTA1–T146) fusion protein or control treatments with empty CTA1R9K-DD vector (mCTA1-empty), CTA1R9K-OVAp323-DD (mCTA1-OVA), all at 5 µg/dose, or peptide alone T146–162 at 0.25 µg/dose, according to preventive (Prev.) or therapeutic (Ther.) protocols. Clinical EAMG score in the indicated groups are given for: **(B)** preventive protocol A (15 doses, from *d* = 0), **(C)** therapeutic protocol A after second TAChR boost (15 doses, from *d* = 60), **(D)** therapeutic protocol B after second TAChR boost (10 doses, from *d* = 60) and **(E)** therapeutic protocol C with clinical scores and **(F)** showing weight curves for **(E)** in mice with treatments starting on *d* = 70 and 10 i.n. doses. *N* = 10 mice/group; data are expressed as means ± SEM and significance was calculated with 2-way-ANOVA analysis plus Bonferroni *post hoc* comparisons. *p*-Values are represented by *p < 0.05, **p < 0.01, and ***p < 0.001.

Next, we extended the analysis to include long-term effects of the mCTA1–T146 fusion protein on the development of EAMG disease. We evaluated different treatment protocols as shown in Figure 2A, and mice were carefully monitored for disease development. Irrespective of whether the fusion protein was administered at an early phase of disease-induction (Figure 2B) or later, during the chronic phase of the disease (Figures 2C–E), we achieved strong suppressive effects of i.n. mCTA1–T146 fusion protein on disease manifestations compared to PBS or treatments with irrelevant fusion proteins. We observed significantly reduced EAMG clinical scores (Figures 2B–E). While disease scores at the end of the experiments were near 2 or above in PBS-treated EAMG mice, disease severity in mCTA1–T146 treated mice was always near or below 1. We also evaluated the clinical efficacy of 10 i.n. doses (therapeutic protocols B and C, Figure 2A), compared to the 15 doses used in the first experiments (preventive protocol A and therapeutic protocol A, Figure 2A), but we found no statistical difference between the two protocols, arguing that both the number of treatments and the total amount of fusion protein could be reduced and still a strong suppressive effect was achieved (Figures 2D–E). The effect of the treatment was also detectable as significantly less weight loss compared to untreated (PBS) EAMG diseased mice using the therapeutic protocol (Figure 2F). Mice treated with a fusion protein without the AChR-peptide (mCTA1-empty, Figure 2B), or with a fusion protein containing an irrelevant control peptide (mCTA1-OVA, Figure 2C), or with equimolar doses of peptide alone (Figure 2D) failed to show any significant effects on disease manifestations.

### Treatment Effects Are Reflected in Suppressed AChR-Specific Antibody Levels and Preserved Muscle AChR Content

**Figure 3:**
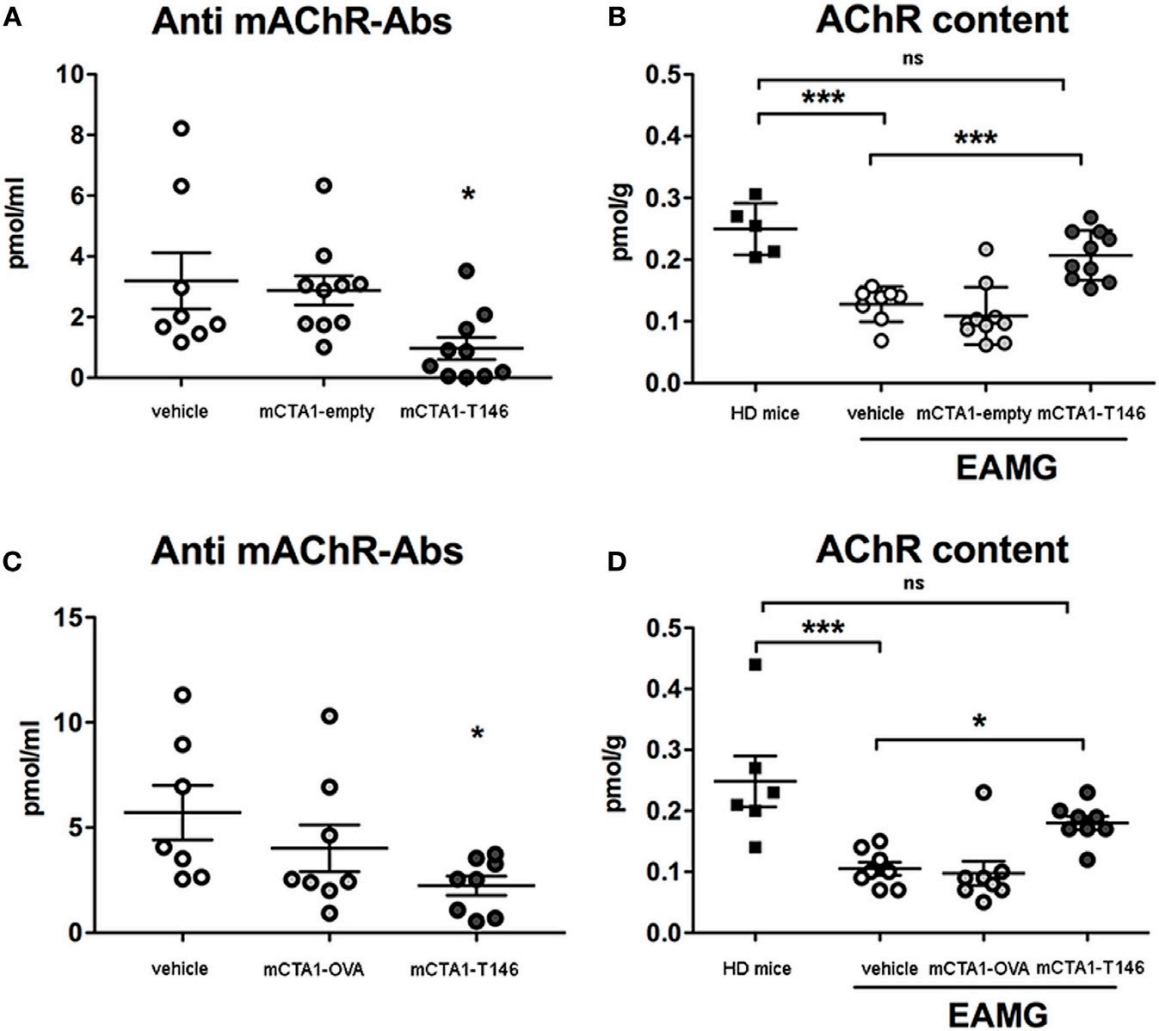
Tolerogenic treatment with mCTA1–T146 protects against loss of muscular acetylcholine receptor (AChR) and impairs anti-AChR-specific serum antibody responses. Anti-AChR serum antibody levels (pmol/ml) in individual mice from Prev. A **(A)** or Ther. A **(C)** protocols used for protection against experimental autoimmune myasthenia gravis (EAMG) disease in mice by i.n. treatment with mCTA1–T146, compared with PBS (vehicle), mCTA1-empty or CTA1R9K-OVA_p323_-DD (mCTA1-OVA). The muscle content of AChR for individual mice was determined using a radioimmunoassay and total muscle AChR content (pmol/g of tissue) is given in healthy donor mice (HD mice) compared to that detected in mice undergoing i.n. tolerizing treatment according to Prev. A **(B)** or Ther. A **(D)** protocols—values are given for individual mice in pmol/ml and means ± SEM of serum antibody levels or AChR muscle content for individual mice in pmol/g and means ± SEM. Significance was calculated with one-way ANOVA analysis plus Dunnet *post hoc* comparisons. *p*-Values are represented by ns, not significant; *p < 0.05; ***p < 0.001.

Experimental autoimmune myasthenia gravis disease is associated with increased anti-AChR antibody levels in serum and loss of muscular AChR content. We found the accompanying increased serum anti-AChR antibody levels in EAMG disease less prominent in mCTA1–T146 treated as opposed to PBS-treated control mice. In fact, a significantly lower anti-AChR serum titer was found in mCTA1–T146-treated as compared to both PBS- or control fusion protein (mCTA1-empty or mCTA1-OVA)-treated mice (Figures 3A,C). We also evaluated AChR content in muscles from treated and untreated EAMG mice: higher levels of muscle AChR content were found in mCTA1–T146-treated EAMG mice compared to PBS- or control fusion protein-treated mice (mCTA1-empty or mCTA1-OVA) (Figures 3B,D). Moreover, the muscular AChR content in mCTA1–T146-treated mice was not significantly lower than that recovered in muscle from healthy mice. This was apparent in both early (Figure 3B) and late (Figure 3D) treatment protocols. Thus, the i.n. treatment with mCTA1–T146 fusion protein prevented from loss of muscle AChR content, and this was associated with a significantly reduced level of anti-AChR serum antibodies.

### Suppression of EAMG Disease Is Associated with Lower IFNγ and IL17 Production and Upregulated Expression of TGFβ, IL10, IL27, and FOXP3 Genes

**Figure 4:**
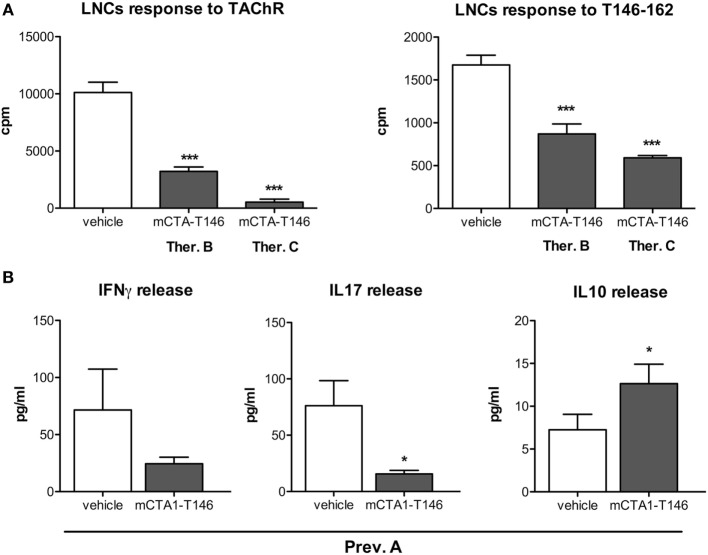
Tolerization with mCTA1–T146 effectively suppresses autoaggressive lymph node T cell functions and promotes regulatory T cells. **(A)** LNCs responses to recall antigen TAChR (0.25 µg/ml) or T146–162 peptide (10 µg/ml) were assessed. Cells were isolated from experimental autoimmune myasthenia gravis mice following i.n. treatment with PBS (vehicle) or the tolerogen CTA1R9K-AChR_146_-DD (mCTA1–T146) as indicated; animals received the therapeutic protocol B (Ther. B), therapeutic protocol C (Ther. C), or preventive protocol A (Prev.A). LNCs proliferation is given as mean cpm values ± SEM. **(B)** Cytokine production in LNCs supernatants; data are expressed as mean pg/ml ± SD. Representative experiments of four, *n* = 10 mice per group; significance was calculated with Mann–Whitney test. *p*-Values are represented by *p < 0.05; ***p < 0.001.

**Figure 5:**
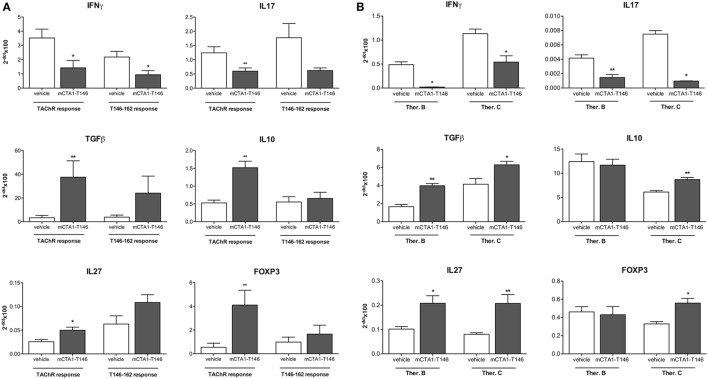
Suppression of genes encoding effector cytokines while upregulating genes encoding tolerance-induction in CD4 T cells after i.n. treatment with the mCTA1–T146 in experimental autoimmune myasthenia gravis (EAMG) mice. mRNA expression of genes encoding the pro-inflammatory cytokines IFNγ and IL17 or regulatory genes encoding TGFβ, IL10, IL27, and Foxp3 were assessed in cultured LNCs **(A)** or spleen **(B)** of tolerogen- (mCTA1–T146) or PBS (vehicle)-treated EAMG mice, as described in Figure 4. Cells were *in vitro* stimulation for 72 h with TAChR (0.25 µg/ml) or T146–162 peptide (25 µg/ml), and mRNA analyzed by qRT-PCR. EAMG mice were treated i.n. with the tolerogen CTA1R9K-AChR_146_-DD (mCTA1–T146) or PBS (vehicle) according to protocol therapeutic B (Ther. B) and C (Ther. C). mRNA values were normalized to the GAPDH housekeeping gene and expressed as mean 2^−Δct^ × 100 ± SEM. Representative experiment of two, *n* = 10 mice per group; significance was calculated with Mann–Whitney test. *p*-Values are represented by *p < 0.05; **p < 0.01.

We evaluated the CD4 T cell responses to recall antigen in the draining lymph nodes derived from mCTA1–T146-treated mice. Results from three different treatment protocols were compared (Prev. A, Ther. B and C). We found significantly impaired CD4^+^ T cell proliferation in draining lymph nodes to both the TAChR and T146–162 peptide (Figures 4A,B, respectively). The cytokine responses induced exhibited substantially reduced IFNγ and IL17 production, while IL10 levels were increased in mCTA1–T146 culture supernatants from treated mice as compared to PBS-treated mice (Ther. A Figures 2 and 4B). Furthermore, the mRNA expression levels of some critical genes were assessed in draining lymph nodes and spleen and compared to those found in PBS-treated animals. Whereas PBS-treated mice exhibited strongly enhanced expression of mRNAs encoding IFNγ and IL17 in lymph node cells, these effector cytokine mRNAs were dramatically reduced in mCTA1–T146-treated mice (Figure 5A). By contrast, mCTA1–T146-treated mice exhibited enhanced TGFβ, IL10, IL27, and Foxp3 mRNA levels compared to those found in PBS-treated mice (Figure 5A). This pattern of gene expression was also observed in splenocytes from treated as opposed to control mice (Figure 5B). Hence, genes encoding TGFβ, IL10, IL27, and FoxP3 cytokines, associated with immune suppression of Th1 and Th17 effector CD4^+^ T cell development, were significantly more expressed in lymph nodes and spleens in mCTA1–T146-treated as opposed to PBS-treated mice. Taken together, our data indicated a strong tolerogenic effect of mCTA1–T146, effectively suppressing autoaggressive effector CD4^+^ T cell functions. Accordingly, it appeared that mCTA1–T146 treatment in EAMG mice promoted immune tolerance by reinstating immunoregulatory CD4^+^ T cells. Both Tregs and Tr1 CD4^+^ T cells could be involved in this tolerance (34). Interestingly, the IL27 pathway, known to promote Tr1 cell differentiation, appears to be involved, which corroborates earlier findings with our tolerogen in the CIA model (Figures 5A,B).

## Discussion

The present study identifies a novel approach to treating MG disease by reinstating tolerance against the AChR using the targeted fusion protein mCTA1–T146. We provide compelling evidence in the mouse EAMG model that i.n. treatment with mCTA1–T146 is effective at an early as well as at a later chronic stage of the disease with significantly less tissue destruction, i.e., higher muscle AChR content, and lower serum anti-AChR antibody titers than in untreated mice. This has important implications for the feasibility of developing a clinical treatment for MG patients. Moreover, the effect on lowering the clinical score was associated with an immune suppressive gene signature involving upregulation of genes encoding for IL10, IL27, TGFβ, and FoxP3 in the draining lymph nodes and spleen. These genes have been found strongly linked to a protective effect on autoimmune reactions (35). Most importantly the production of AChR-specific IFNγ and IL17, key cytokines representing Th1 and Th17 functions, respectively, were significantly suppressed in mCTA1–T146-treated mice. In this way, the present findings corroborate and extend our previous documentation of a Tr1-mediated suppressive effect of the fusion protein on autoaggressive CD4^+^ T cells in the CIA model (26).

The pioneering experiments showing that mucosal administration of native TAChR in rats induces tolerance dates back to the early 1990s when nasal or oral delivery was shown to suppress EAMG disease (29, 36, 37). Since then, several studies have been published that convincingly demonstrate that mucosal delivery of AChR or its recombinant extracellular α1 domain effectively suppresses induction of EAMG in both the rat and mouse EAMG models (38). Notably, it was observed that in ongoing EAMG it was significantly more difficult to suppress the disease and it usually, but not always, required much larger doses of antigen (19, 39). Also peptides have been used for tolerization and it has been well documented that most often large doses (50–100 µg or more) are required for suppressing induction of EAMG disease (29, 40, 41). It is in this context, we believe our targeted fusion protein, mCTA1–T146, adds to the therapeutic arsenal of AChR-specific tolerance-inducing strategies.

We found that repeated nasal treatments with a low dose of mCTA1–T146 effectively ameliorated EAMG disease resulting in less reduction in muscle AChR content and lower levels of serum anti-AChR antibodies. Because the peptide T146–162 is incorporated into the fusion protein, it has to be taken up and be processed by antigen-presenting cells (APC) to induce tolerance (27). This is contrary to the peptide approach where uptake by APC and processing are not involved and, indeed, the term apitope has been coined to identify an antigen-processing independent epitope (42–44). Therefore, the mechanisms of action can potentially be different between a peptide approach and that of the fusion protein. In particular, lower molar doses would be expected to be required for a tolerance-inducing effect by the fusion protein, which is also supported in our study when comparing the fusion protein with an equimolar dose of peptide that failed to induce tolerance. However, in the experimental autoimmune encephalomyelitis (EAE) model, apitope-induced tolerance was also mediated by IL-10 producing Foxp3-negative Tr1 cells, similar to what we think our mCTA1–T146 fusion protein does in the EAMG model (45). Nevertheless, we believe the fusion protein approach may have certain merits as it is possible to attach targeting elements to the peptides. In the mCTA1–T146 fusion protein, we have explored the DD-moiety for its DC targeting ability. Given i.n. the migratory DCs of the CD103^+^ phenotype are the primary targets for inducing tolerance (46, 47). Indeed, in preliminary experiments we could also show that our fusion protein accumulated in CD103^+^ migratory DC following i.n. administration (C. Hansson, Univ. of Gothenburg, Sweden, personal communication).

The critical disease-preventing element in the fusion protein is the AChR-specific peptide T146–162. Hence, the immune system in general is, therefore, unperturbed and functional for protection against, e.g., pathogens. Moreover while the fusion protein is carrying the T146–162 peptide, rather than the whole extracellular α1 of AChR, i.e., aa 1–210, the peptide approach largely avoids an immunogenic and potentially pathological reactivity to B cell epitopes in the protein (3). Hence, this also limits the risk of disease exacerbation following treatment should a enhancing rather than a suppressive anti-AChR-specific antibody response be elicited, including potential epitopes due to renaturation of conformation-dependent epitopes (3, 39, 48, 49). This is also the reason why investigators have avoided the main immunogenic region (i.e., aa 66–76) loop when developing α1 region based tolerogenic treatments and why cytoplasmatic domains rather than the extracellular α1 region of the AChR are being used for tolerance induction (3, 50). However, it appears that rather large doses are required for tolerance induction using this approach, at least in the rat EAMG model (48).

According to the guidelines for the mouse EAMG model, the quality of the TAChR preparation is important to get a high take of disease (21). In this study, we achieved 95% incidence of disease. Although, our preparation could have contained low levels of contaminating other synaptic proteins (see Materials and Methods), we specifically tolerized against the T146 peptide of the AChR, and, hence, we suppressed CD4 T cell reactivity against the AChR with positive clinical manifestations. However, additional incorporated peptides, representing multiple synaptic proteins, may have improved suppression even further. It has been debated whether a single or few peptides from a given protein will suffice in the clinic to reinstate tolerance (44). One argument against the use of a single peptide has been that since MG patients usually recognize multiple epitopes in AChR, it will be difficult to reinstate tolerance in multiple CD4 T cell clones with diverse epitope recognition (3). Given that many AChR-epitopes are dominant and probably do not host B cell epitopes, it would be feasible to combine several of these epitopes in a fusion protein (49, 51). This would also be a mechanism to broaden the scope of peptide-binding to surface molecules encoded by the HLA-D-locus in a genetically diverse population, although the 146–162 amino acid peptide has proven to bind to quite a large and diverse range of HLA-DQ subset of haplotypes prevalent in Caucasians as well as in Afro-Americans and Asians (52).

Some researchers have investigated the concept of a therapeutic intervention by reinstating Tregs in MG patients (9). Whereas the technology appears clinically cumbersome, costly and possibly associated with significant risk it may not be a feasible future therapeutic approach. The idea that polyclonal autologous inducible Tregs (iTregs) expanded *ex vivo* and adoptively transferred to the patient suffers from the inconsistency that also other immune responses will be suppressed in these patients, potentially placing these patients at risk of acquiring infectious diseases or developing cancer. An alternative strategy to expanding Tregs is to transfer autologous immature or tolerogenic DCs prior to or subsequent to inducing EAMG (53, 54). In this way, Tregs will expand *in vivo* and suppress disease. These *ex vivo* generated DC could be treated with TGFβ, IL10, or substances that promote a tolerogenic phenotype like rapamycin or statin (11, 55, 56). However, even though this strategy seemed promising in the experimental EAMG model its clinical application appears unrealistic. By contrast, the fusion protein would achieve the induction of Tregs *in vivo* and in this way circumvent *ex vivo* expansion and handling of Tregs.

Our mCTA1–T146 fusion protein acts through suppressing the autoaggressive Th1 and Th17 cells directed against epitopes in the AChR. As aforementioned the mechanism of action is through induction of regulatory CD4^+^ T cells, of which both Foxp3^+^ Tregs and Tr1 cells appeared to be induced in the present study. Although we found upregulated Foxp3-gene expression in the present study, we have previously observed clear evidence of an IL-10 dependent mechanism associated with Foxp3^−^ CD4 T cells in the CIA model (26). Recently, we have further strengthened a Tr1-mediated mechanism by showing that CD49b^+^LAG3^+^ CD4 T cells were induced in the draining lymph node after i.n. immunizations with our tolerogen for treatment of the EAE mouse model (C. Hansson, Univ. of Gothenburg, Sweden, unpublished observation) (57). Moreover, the same suppressive gene signature with upregulated gene expression for IL10, IL27, TGFβ, and Foxp3 was observed also in this latter model (45, 58–61). The upregulated Foxp3-gene expression could, however, indicate that also iTregs are induced and participate in the suppression of AChR autoreactive CD4^+^ T cells in treated mice. Perhaps, induction of both Tr1 and iTregs is the more likely scenario to explain the effective suppressive function of mCTA1–T146 in the EAMG model. Contrary to the acute CIA model the EAMG model has a chronic ongoing phase of disease (21, 33). It is promising to note that our tolerogen was equally effective in the late chronic phase of EAMG as in the early phase of disease development. We have previously demonstrated that oral administration of the Tα146–162 synthetic peptide in milligram doses to mice reduced the T-cell proliferative response to TAChR or Tα146–162 peptide and peptide doses given i.n. in 100 µg could achieve a similar effect (29). Hence, the mCTA1–T146 fusion protein is significantly more effective and requires much lower and fewer doses (a total dose of 50 µg of mCTA1–T146) to substantially reduce ongoing EAMG than peptide alone or derivatives of AChR. Ongoing studies aim to define the minimal protocol for significant improvement of EAMG disease in the mouse model using mCTA1–T146. The present study clearly demonstrates the effectiveness of the targeted mCTA1–T146 fusion protein and helps identify a new promising strategy in tolerance-inducing treatments of autoimmune diseases in general, and of MG-disease, in particular.

## Materials and Methods

### Animals

Female 6- to 8-week-old C57BL6/N mice were purchased from Charles River Laboratories Italia (Calco, Italy) and housed at the animal facility of the Institute. Most experimental groups hosted 10 animals per group and more than 100 mice were tested for reinstatement of tolerance with the CTA1R9K-AChR_146–162_-DD fusion protein. The group size was determined according to the protocol by Snedecore and Cochran for significance and mean disease severity in C57Bl/6 mice (62). All procedures involving animals were approved by the Institute Ethical Board and Italian Ministry of Health (1064/2015-PR) and were performed according to the Italian Principle of Laboratory Animal Care (DDL 116/92 and DLgs 26/2014), and the European Communities Council Directive 86/609/EEC and 2010/63/UE. Animals were sacrificed after deep anesthesia induced by carbon dioxide. Low-grade anesthesia with 2% isoflurane (60:40 N_2_O:O_2_, flow rate 0.8 l/min) was induced in animals prior to immunizations and treatments.

### Preparation of Fusion Proteins

The fusion protein CTA1R9K-DD, with or without one copy of OVA_323–339_ peptide (CTA1/R9K-OVA-DD) or AChR_146–162_ peptide (CTA1R9K-AChR_146_-DD) with the torpedo amino acid sequence LGIWTYDGTKVSISPES were prepared as previously described (63). Briefly, for expression and purification, *Escherichia coli* BL-21 cells transformed with the different expression plasmids were grown in 2× YT medium with 50 mg/ml Kanamycin at 37°C overnight. Fusion proteins were produced as inclusion bodies and solubilized using 6 M guanidine-HCl. After refolding in distilled water, the fusion proteins were purified by affinity chromatography on IgG Sepharose (Pharmacia) as described, and stored in PBS at −80°C. Endotoxin levels were low (<10 EU endotoxin/mg protein) as measured by end point chromogenic limulus amebocyte lysate methods (LAL Endochrome™, Charles River Endosafe, Charleston, SC, USA). The preparations were tested for not having any ADP-ribosylating activity using the Agmatine assay as described (26).

### EAMG Experimental Model and Treatment Protocols

The EAMG model was performed essentially as described in the guidelines, except for testing the purification of AChR by rigorous gel electrophoresis and assessing possible contaminating synaptic proteins (see below) (21). Briefly, experimental TAChR-EAMG (21, 64) was induced by s.c. immunizations in the hind limbs (multiple sites) with 20 µg of purified TAChR (from *Torpedo californica* electric organ; Aquatic Research Consultants) emulsified 1:1 ratio in Complete Freund’s Adjuvant (CFA; Difco), in a total volume of 200 µl. After the priming immunization, two subsequent TAChR boosts (20 µg of TAChR emulsified in incomplete Freund’s adjuvant in a total volume of 200 µl/mouse) were given on day 30 and 60 to induce EAMG. The clinical scores and weight loss were measured by blinded investigators, ignorant about individual treatments of the mice. Experimental groups consisted of 10 mice, unless otherwise stated in the figure legends. Spleen, inguinal, and popliteal lymph nodes were taken and blood was collected and immediately processed pending further analyses. EAMG mice were treated i.n. with 5 µg of fusion protein.

### TAChR Preparation

TAChR was purified from *Torpedo californica* electric organ according to the alkali-stripped membrane protocol (65, 66), with minor modifications. Briefly, the electric organ tissue was homogenized in 10 mM sodium phosphate buffer, 1 mM EDTA, 0.02% NaN_3_, 0.01 mM PMSF, pH 7.8 for 3 min, high speed. The extract was centrifuged for 1 h at 100,000 × *g*. The pellet was resuspended in ice-cold water and the pH adjusted to 11.0 with NaOH; the membranes were immediately centrifuged for 30 min at 100,000 × *g*. TAChR was solubilized from membranes with 2% sodium deoxycholate, overnight at 4°C, then centrifuged at 100,000 × *g* for 1 h. TAChR concentration was determined as α-BTX (bungarotoxin)-binding sites/milliliter and protein concentration by the BCA Protein Assay Kit (Thermo Scientific). Sodium deoxycholate was partially removed by progressive dialysis (1%, and then 0.05%), and TAChR aliquots were stored at −80°C. The average activity of the TAChR preparation corresponded to 0.87–1.1 nmol of ^125^I-labeled-αBTX-binding sites/milligram of protein, which is in line with the separation of membrane molecules by sucrose-gradient centrifugation, as described by Elliott et al. (66). In the final preparation the estimated TAChR concentration was 487 µg/ml and the total protein content was 2,000 µg/ml. Of note, the biological activity of TAChR was evaluated as the number of α-BTX-binding sites/milligram of protein, as reported in the published guidelines for the mouse EAMG model (21). This is an assay reflecting the integrity of the native AChR conformation, with organized α, β, δ, and γ subunits, functionally binding to α-BTX. A gel electrophoresis also identified the four subunits. However, a more rigorous gel electrophoresis of the preparation was not undertaken; hence, we cannot exclude that also other potential contaminating synaptic proteins were present. We applied all precautions and safety measures recommended by the manufacturer when working with BTX.

### EAMG Clinical Evaluation

Each animal was weighed and scored at the beginning of each experiment and at least twice weekly until the end of the experiment; clinical scores were taken every 24 h or more often if the animals demonstrated severe disease (21). EAMG clinical score was assessed after exercise for 30 s, using the grip strength test. Disease severity was graded as follows: grade 0, normal strength and no abnormalities; grade 1, mildly decreased activity and weak grip or cry; grade 2, clinical signs present before exercise (tremor, head down, hunched posture, weak grip); grade 3, severe clinical signs at rest, no grip, moribund; grade 4, sacrifice, humane end point. EAMG was confirmed by i.p. injections of Prostigmine. Animals were sacrificed 10–12 weeks after TAChR/CFA immunizations.

### Cell Proliferation

Recall antigen responses were assessed in single cell suspensions from draining (popliteal and inguinal) lymph nodes from EAMG mice after i.n. treatment with fusion protein or PBS. Cells were cultured in quadruplicates in 96-well plates at 2 × 10^5^ cells/well with 0.25 µg/ml TAChR or with 10 µg/ml T146–162 peptide (LGIWTYDGTKVSISPES); concanavalin A (ConA, 2 µg/ml, Sigma) was used as positive control. The RPMI culture medium (Euroclone) was supplemented with 10% FCS, 1% Na-pyruvate, 1% non-essential aa, 1% l-glutamine, 1% penicillin–streptomycin (Euroclone), 50 µM 2-mercaptoethanol (Sigma), plus 1% normal rat serum. After 72 h of incubation at 37°C and 5% CO_2_, the cultures were pulsed with 0.5 µCi [^3^H]-thymidine/well for 18 h, and cell proliferation was measured using a beta counter (PerkinElmer).

### Muscle AChR Content

Mouse AChR was extracted from the whole mouse carcass. Each carcass was weighed and homogenized for 1 min at high speed in four volumes of homogenization buffer (0.1 M NaCl, 10 mM NaN_3_, 0.01 M EDTA, 0.01 M EGTA, 0.01 M iodoacetamide, 1 mM PMSF, 1 mM sodium phosphate buffer, pH 7.5). The homogenized extract was centrifuged 30 min at 17,000 × *g* and pellet was homogenized for 1 min at high speed in one volume of homogenization buffer. AChR was solubilized from membranes with Triton X-100 (10%) in Tris buffer, for 4 h at 4°C, then centrifuged at 100,000 × *g* for 30 min (4°C). Duplicate 0.1 ml aliquots of mouse muscle AChR crude extract were incubated with an excess of [^125^I]α-BTX, and transferred to a DE-81 DEAE disk followed by washing with Triton X-100 (0.5%) Tris buffer. Radioactivity was determined by gamma counting. Unspecific binding (from parallel tubes pre-incubated with unlabeled α-BTX) was subtracted from each sample. Results were expressed as picomoles of toxin-binding sites per gram of carcass.

### Anti-AChR Antibody Titer

Acetylcholine receptor-specific antibodies were determined in serum from individual mice by a radioimmunoprecipitation assay (67). Mouse AChR was extracted from whole carcass, as previously described, and labeled with 2 nM [^125^I]α-BTX. Serum samples were incubated overnight with [^125^I]-αBTX labeled mouse AChR (0.5 pM). Antibody–AChR complexes were precipitated by adding an excess of rabbit anti-mouse IgG (DAKO). Pellets were washed twice with cold PBS plus 0.5% Triton X-100 (Carlo Erba) and [^125^I]-αBTX labeling was assessed using a γ-counter (PerkinElmer). Serum samples incubated with mouse AChR and pre-incubated in excess of unlabeled α-BTX (Life Technologies) (unspecific binding) were subtracted from test samples. The anti-AChR antibody titers were expressed as pM of [^125^I]α-BTX-binding sites precipitated per milliliter of serum.

### cDNA Synthesis and qPCR

Total RNA was extracted from the draining lymph nodes using the Trizol reagent; cDNA was synthesized from RNA using random hexamers and reverse transcriptase (all from Life Technologies). Real-time quantitative PCR for IFNγ, IL17, TGFβ, IL10, IL27, FoxP3 was performed using Assay-on Demand Gene Expression Products (Life Technologies). GAPDH was used as housekeeping endogenous genes. mRNA levels of target genes were expressed as relative values (2^−Δct^ × 100) normalized toward the chosen housekeeping genes, in which ΔCt represents the difference between cycle threshold (Ct) of the target gene and Ct of the housekeeping gene. Real-time PCR reactions were performed in duplicates using an ABI Prism 7500 FAST Real-Time PCR System (Life Technologies).

### Multiple Cytokine Assay

Culture supernatants were analyzed for IFNγ, IL17, and IL10 cytokine expression by ProcartaPlex Mouse IFNγ, IL17A, and IL10 Simplex kits (Affymetrix—eBioscience), according to the manufacturer’s instructions. The limit for detection of individual cytokines was 2–5 pg/ml. Plates were read in a Luminex MAGPIX instrument (Luminex Corporation). Analysis of data and quantification of cytokines was performed using the Luminex xPONENT Software (Luminex Corporation) on the basis of corresponding standards curves.

### Statistical Analysis

Results were statistically analyzed using GraphPad Prism v5.0 (GraphPad Prism, CA, USA) and values were given as means ± SEM or SD, as indicated. Statistical analysis was performed according to the nature of data. Normally distributed data were analyzed using one- or two-way ANOVA followed by appropriate *post hoc* comparisons. Non-parametrically distributed data were analyzed using the Mann–Whitney test. Statistical significance is given as *p*-values: *p < 0.05, **p < 0.01, and ***p < 0.001.

## Ethics Statement

This study was carried out in accordance with the recommendations of the Institute Ethical Board and Italian Ministry of Health (1064/2015-PR) and were performed in respect to the Italian Principle of Laboratory Animal Care (DDL 116/92 and DLgs 26/2014), and in accordance to European Communities Council Directive 86/609/EEC and 2010/63/UE.

## Author Contributions

AC and ER performed the animal experiments. AC, SS, KS, and CL-F analyzed the data and interpreted the results. AC and CL-F prepared the figures. NL and FB designed the experiments. AC, NL, and FB wrote the manuscript.

## Conflict of Interest Statement

NL has intellectual property related to MG treatment. NL is affiliated with the Toleranzia AB company that develops MG-specific treatments based on the fusion protein described in the present study. Disclosures are managed in compliance with the policies of University of Gothenburg, Sweden. AC, SS, KS, CL-F, ER, and FB have nothing to disclose.
